# Eleven-year retrospective study characterizing patients with severe brain damage and poor neurological prognosis -role of physicians’ attitude toward life-sustaining treatment

**DOI:** 10.1186/s12904-022-00975-8

**Published:** 2022-05-18

**Authors:** Haruaki Wakatake, Koichi Hayashi, Yuka Kitano, Hsiang-Chin Hsu, Toru Yoshida, Yoshihiro Masui, Yasuhiko Taira, Shigeki Fujitani

**Affiliations:** 1 Department of Emergency and Critical Care Medicine, St. Marianna University, Yokohama Seibu Hospital, 1197-1 Yasashi-cho, Asahi-ku, Yokohama, Kanagawa 241-0811 Japan; 2 Department of Emergency Medicine, College of Medicine, National Cheng Kung University Hospital, National Cheng Kung University, 138 Sheng-Li Road, Tainan, 70428 Taiwan; 3 Department of Emergency and Critical Care Medicine, St. Marianna University School of Medicine, 2-16-1 Sugano, Kawasaki, Kanagawa 216-8511 Japan

**Keywords:** Brain hemorrhage, Cerebral infarction, Cardiac arrest, Brain death, Withdrawal, Attitude toward treatment, Ethics

## Abstract

**Background:**

Severe brain hemorrhage/infarction and cardiac arrest constitute the most critical situations leading to poor neurological prognosis. Characterization of these patients is required to offer successful end-of-life care, but actual practice is affected by multiple confounding factors, including ethicolegal issues, particular in Japan and Asia. The aim of this study is to evaluate the clinical courses of patients with severe brain damage and to assess the preference of end-of-life care for these patients in Japanese hospitals.

**Methods:**

A retrospective observational study was conducted between 2008 and 2018. All intracranial hemorrhage/infarction and cardiac arrest out-patients (*n* = 510) who were admitted to our two affiliated hospitals and survived but with poor neurologic outcomes were included. Demographic characteristics as well as prognosis and treatment policies were also assessed.

**Results:**

Patients were divided into two categories; cases with absent brainstem reflex (BSR) (BSR[-]) and those with preserved BSR (BSR[ +]). The survival rate was higher and the length of hospitalization was longer in patients with BSR[ +] than in those with BSR[-]. Among three life-sustaining policies (i.e., aggressive treatment, withdrawal of treatment, and withholding of treatment), withholding of treatment was adopted to most patients. In BSR[-], the proportion of three treatment policies performed at the final decision did not differ from that at the initial diagnosis on neurological status (*p* = 0.432). In contrast, this proportion tended to be altered in BSR[ +] (*p* = 0.072), with a decreasing tendency of aggressive treatment and a modest increasing tendency of withdrawal of treatment. Furthermore, the requests from patients’ families to withdraw life-sustaining treatment, including discontinuation of mechanical ventilation, increased, but actual implementation of withdrawal by physicians was less than half of the requests.

**Conclusions:**

BSR constitutes a crucial determinant of mortality and length of hospitalization in comatose patients with severe brain damage. Although the number of withdrawal of life-sustaining treatment tends to increase over time in BSR[ +] patients, there are many more requests from patients’ families for withdrawal. Since physicians has a tendency to desist from withdrawing life-sustaining treatment, more in-depth communication between medical staff and patients’ families will facilitate mutual understanding over ethicolegal and religious issues and may thus improve end-of-life care.

## Background

Severe brain damage with poor neurological prognosis is a critical state that reflects devastated conditions, including brain death, brainstem dysfunction and irreversible hypoxic encephalopathy. Cardiopulmonary arrest (CPA) is one of the most crucial events and constitutes a major leading cause of death, accounting for more than 10,000 cases annually in Japan [1]. Only 20% of out-of-hospital cardiac arrest (OHCA) patients are able to restore spontaneous systemic circulation, among whom, however, 80% have severe brain damage with poor neurological prognosis [2]. Furthermore, severe brain damage is also precipitated by intracranial disease such as cerebral/subarachnoid hemorrhage and cerebral infarction, which occur in approximately 300,000 subjects annually in Japan [3]. Despite vigorous implementation of medical care, current advances in clinical practice fail to halt the ravaging process or the progression to brain death and brainstem dysfunction in many patients resuscitated from CPA or with critical brain hemorrhage/infarction.

Although advanced brain damage frequently involves ethical considerations or arguments on end-of-life care, the strategy for the medical practice in patients with poor neurological prognosis may vary depending on personal attitudes of physicians or specific regions of the hospitals [4–10]. Most Western physicians recognize that withholding and withdrawal of life-sustaining treatment are acceptable [4–7, 10]. In contrast, Asian countries are facing different views regarding physicians’ attitudes toward end-of-life care [4, 5, 8, 10]. In Japan, physicians used to be reluctant to withdraw life-sustaining treatment for patients who were comatose and had poor neurological prognosis [4, 5]. Meanwhile, the Government [11] and several academic societies [12–14] published the guidelines for end-of-life care, which qualified the physicians’ attitudes toward withdrawal of life-sustaining treatment. Under the milieu where medical care becomes more complicated and multifaceted, however, much concern arises regarding availability and necessity of life-sustaining measures, post-resuscitation care and prolonged hospitalization, all of which entail important issues to be settled with utmost requirement. Thus, there still remains a controversy as to the decision-making on the end-of-life care and management of the comatose patients with severe brain damage. Furthermore, although the presence of brainstem dysfunction is generally recognized as more severe brain injury [15], it remains undetermined whether the disappearance of brainstem reflex (BSR) modifies the attitudes of physicians toward end-of-life care.

Our medical facilities, i.e., St. Marianna University Hospital and St. Marianna University Yokohama Seibu Hospital, play a central role in the emergency care and management as tertiary medical centers over the Yokohama and Kawasaki area and accept a large number of critically ill patients. During these eleven years, we have experienced many patients with severe brain hemorrhage/infarction or comatose patients who survive from CPA. Among these patients, approximately 500 patients were afflicted with neurologically critical sequelae presenting variable degrees of brain/brainstem dysfunction but not reaching a level of brain death. The purpose of this study is to evaluate the consequence of these patients with special reference to BSR and prognosis. Furthermore, serial changes in the preference of end-of-life treatment, i.e., withdrawal/withholding of treatment, were assessed in Japanese patients with severe brain damage.

## Methods

### Study population

This study, a retrospective observational investigation, was conducted from April 2008 to December 2018 in two affiliated facilities, i.e., St. Marianna University Hospital (Kawasaki) and St. Marianna University Yokohama Seibu Hospital (Yokohama). Both of these hospitals are assigned as tertiary metropolitan medical centers with 1175/518 inpatient beds and comprise 10/10 intensive care unit (ICU) beds and 20/30 high care unit (HCU) beds, respectively. The study was approved by the Institutional Review Board and Ethics Committee of St. Marianna University School of Medicine with waiver of the requirement for obtaining informed consent (institutional approval No. 1614) and was conducted in accordance with the Declaration of Helsinki. Patient information was fetched from electronic medical records and was anonymized prior to final analyses. The study was registered at UMIN (UMIN 000,045,286).

**Fig. 1:**
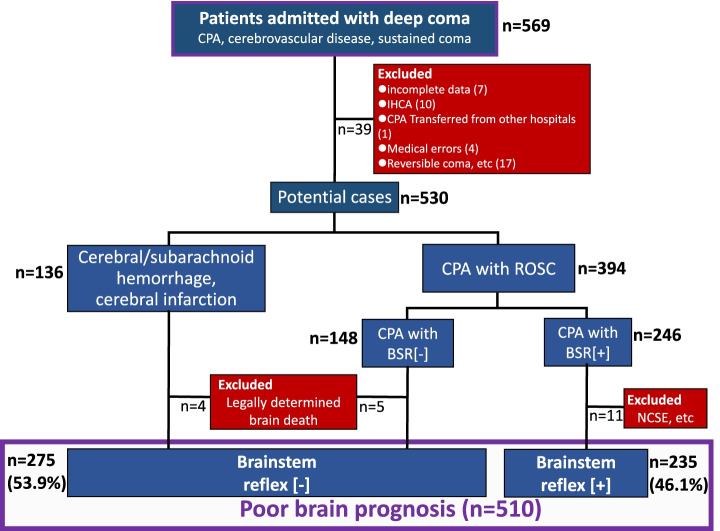
Patient selection. *CPA* Cardiopulmonary arrest, *IHCA* In-hospital cardiac arrest, *ROSC* Return of spontaneous circulation. *NCSE* Non-convulsive status epileptics

The patients devoid of BSR who suffered brain hemorrhage/infarction and severe neurological prognosis or the OHCA patients who survived for more than 72 h but with poor neurologic outcomes were admitted to our hospitals (*n* = 569, Fig. 1). Patients who experienced CPA in our hospitals (i.e., in-hospital CPA) or who had suffered CPA in other hospitals and then transferred to our facilities were not enrolled in this study. In addition, the patients who met the following criteria were excluded; (1) patients in a potentially reversible coma (e.g., acute alcohol/drug addiction, hypothermia, sepsis, uremia, encephalopathy due to metabolic and endocrine diseases, use of neuromuscular blocking or high-dose sedative agents), (2) children under the age of 16, and (3) patients with a history of medical errors or social problems.

Among the potential cases (*n* = 530), the patients who were legally determined as brain death were excluded from the study (Fig. 1). The diagnosis of legally determined brain death was made based on the Japanese guidelines for organ transplantation [https://www.mhlw.go.jp/stf/seisakunitsuite/bunya/0000040046.html] (i.e., flat EEG, absent BSR and positive apnea test). The patients with non-convulsive status epilepticus (NCSE) were excluded from the group of CPA patients. Accordingly, the patients finally enrolled in this study were arbitrarily categorized into two groups; (1) cases devoid of BSR (BSR[-], *n* = 275) and (2) those with preserved BSR (BSR[ +], *n* = 235, Fig. 1).

Demographic data, including age, gender and etiology, were evaluated. Vital signs and neurological status (e.g., BSR, consciousness level, apnea test and EEG data) along with clinical outcomes were also assessed. Prognosis of the patients (e.g., survival to hospital discharge or transfer) and length of stay (LOS) in ICU or hospital were also evaluated.

Upon admission, patients received multipronged life-sustaining therapies, including administration of catecholamines/antibiotics, enteral feeding and endotracheal intubation. The evaluation of the severity of brain damage and the prognostication was performed among the patients who survived more than 72 h and manifested consciousness level of M3 or lower under no influence of sedatives/hypnotics/analgesics [16, 17]. Brain CT was performed in all patients and approximately half of the patients received EEG.

Following the assessment of patients’ neurological status and prognostication, treatment policies were repeatedly discussed with their families/surrogates, i.e., whether they should receive aggressive life-sustaining support therapy or whether ongoing life-sustaining therapy should be withdrawn/withheld. We also obtained from the families the information regarding the perception about end-of-life care that the patients had embraced. Based on the results of the in-depth discussion, final therapeutic policies were determined by multiple physicians, including neurologists.

### Statistical analysis

Results of continuous variables are expressed as the median (interquartile range; IQR). Categorical variables are presented as the number. The Mann–Whitney U test or the Kruskal–Wallis test was used for comparison between two groups or among three groups, respectively. The chi-square test or Fisher’s exact test was applied for analysis of categorical valuables. Statistical analyses were performed using IBM SPSS Statistics for Windows, Version 25 (IBM Japan Ltd, Japan). P values less than 0.05 were considered statistically significant.

## Results

**Fig. 2:**
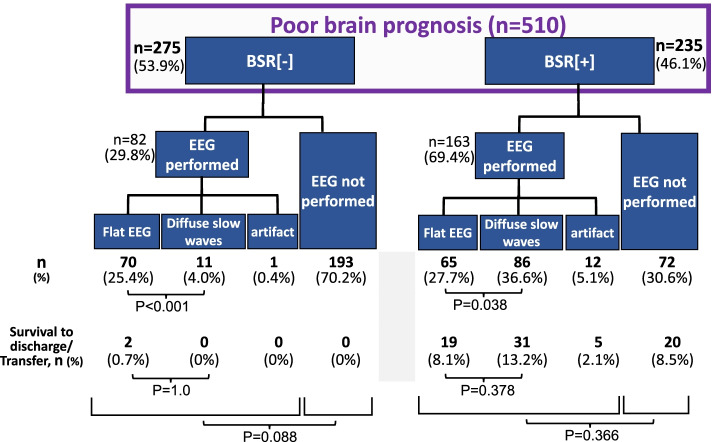
Patients’ distribution and EEG findings. EEG was performed in 29.8% of the patients with brainstem reflex [-] and 69.4% of those with brainstem reflex [ +]. In the subgroup with brainstem reflex [-], paucity of survivors failed to demonstrate the specific role of EEG implementation (*p* = 0.088) nor its findings (*p* = 1.0) in the prognosis of the patients

A. ***Patients’ characteristics***
  During the period between April 2008 and December 2018, we enrolled 510 patients with BSR[-] (*n* = 275) and BSR[ +] (*n* = 235) in our two affiliated hospitals (Table 1). The patients with BSR[-] were younger than those with BSR[ +]. As for the etiology, cerebral infarction and intracranial hemorrhage, including subarachnoid and cerebral hemorrhage, accounted for 64.7% of the BSR[-] group whereas 78.7% of the BSR[ +] group was attributed to cardiovascular and respiratory diseases.
B. ***Prognosis and outcomes***
  The survival rate was markedly higher in patients with BSR[ +] than with BSR[-] (31.9% vs. 0.7%, *p* < 0.001, Table 2). Likewise, total period of hospitalization was longer in patients with BSR[ +] (16 vs. 3 days, *p* < 0.001) although LOS in ICU was not different between BSR[-] and BSR[ +]. When evaluated based on the treatment policy, the patients with BSR[ +] receiving aggressive life-sustaining treatment had a longer period of hospitalization (42 days) than those in whom life-sustaining treatment was withheld (13 days, *p* < 0.001). Furthermore, 26.3% (i.e., 51/194) of the group in which life-sustaining therapy was withheld were transferred to nursing hospitals, and the LOS of this subgroup was markedly longer than that of the patients who died during hospitalization (42 vs. 8 days, *p* < 0.001).
  Among 275 patients with BSR[-], only 82 cases (i.e., 29.8%) underwent EEG, whereas 69.4% of the patients with BSR[ +] received EEG examination (*p* < 0.001, Fig. 2). In patients with BSR[-], a flat EEG pattern predominated (25.4%, *p* < 0.001) and diffuse slow waves were observed in 4.0% of the subgroup. Among 235 patients with BSR[ +], diffuse slow waves were more prevalent than flat EEG waves (86 vs. 65 patients, *p* = 0.038).
  At the time of admission, brain CT scan was also performed in all cases, showing that most of the CPA patients manifested diffuse anoxic brain injury. Among the cases with equivocal CT findings, we confirmed the findings of diffuse anoxic brain injury by a second series of CT scan, which was apparent even in patients with no EEG evaluation or indeterminate EEG findings.
  In patients with BSR[-], the survival rate was not different between the subgroup with flat EEG and that with diffuse slow waves (*p* = 1.0, Fig. 2). Similar results were observed in the BSR[ +] group (*p* = 0.378). Interestingly, the survival rate was not dependent on whether EEG was implemented or not in patients with BSR[-] (*p* = 0.088) and BSR[ +] (*p* = 0.366).
C. ***Temporal changes in treatment policy***

**Table 1:**
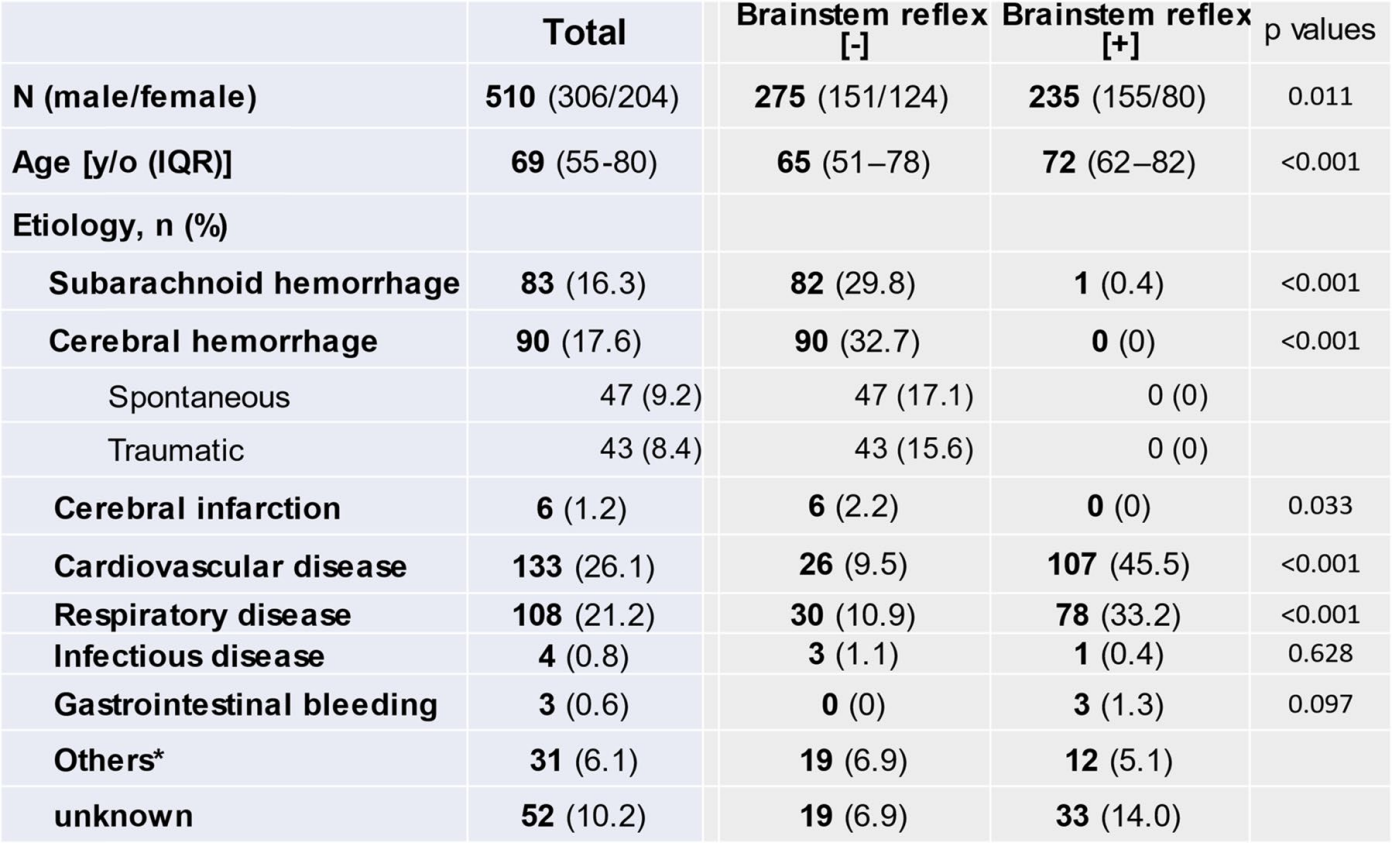
Patients’ characteristics

*; Miscellaneous disorders, Including trauma and Massive systemic hemorrhage

*IQR* Interquartile range

**Table 2:**
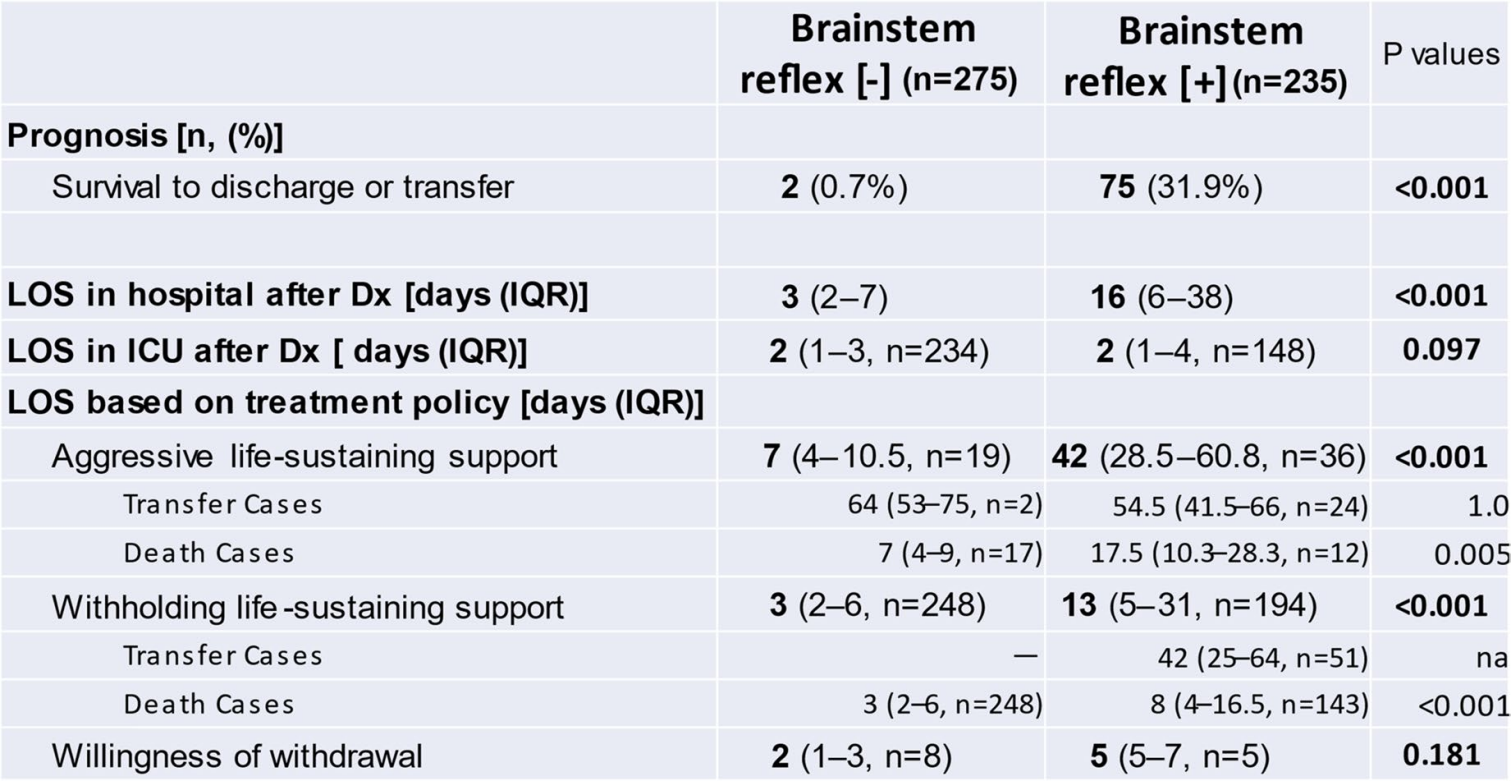
Prognosis and length of hospitalization in patients with brainstem reflex [-]/[ +]

*LOS* Length of stay, *Dx* Diagnosis, *IQR* Interquartile range, *na* Not available

**Fig. 3:**
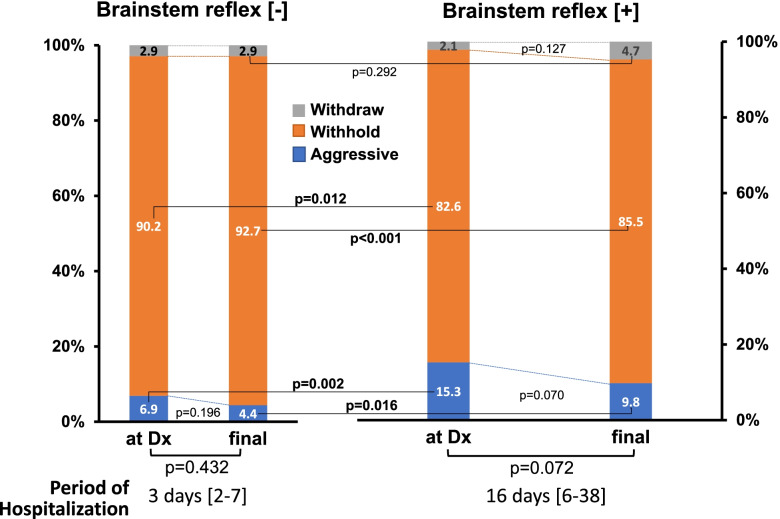
Temporal changes in treatment policies in patients with brainstem reflex [-]/[ +]. At the time of diagnosis (Dx) on patients’ neurological status, a large number of the patients were those whose families wished to withhold life-sustaining treatment in both brainstem reflex [-] and [ +] groups. In patients with brainstem reflex[-], the ratio of three treatment policies (i.e., aggressive treatment, withholding, withdrawal) conducted after the final decision did not differ from that requested at the time of Dx (left). In the brainstem reflex[ +] group, however, the proportion of the treatment policies tended to be altered during 16 [6–38] days (median, IQR), with a decreasing tendency of the aggressive life-sustaining treatment (right)

Figure 3 illustrates the comparison of various treatment policies between BSR[-] and BSR[ +]. At the time of diagnosis on neurological status, a large number of the patients were those whose families wished to withhold life-sustaining treatment although the percentage in the BSR[ +] group was less than that in the BSR[-] group (82.6% vs. 90.2%, *p* = 0.012). Alternatively, the rate of the patients with BSR[ +] receiving aggressive life support was twice as many as that with BSR[-] (15.3% vs 6.9%, *p* = 0.002). The rate of the patients whose families wished to withdraw life-sustaining treatment was nearly the same in the BSR[-] and the BSR[ +] group (2.9% vs. 2.1%, *p* = 0.577).

Temporal changes in the ratio of each treatment policy were assessed. In patients with BSR[-], the proportion of three treatment practices conducted after the final decision did not differ from that of policies decided at the time of diagnosis (χ^2^ test; *p* = 0.432) (Fig. 3). In patients with BSR[ +], the proportion of the treatment policies tended to be altered (χ^2^ test; *p* = 0.072), with a decreasing tendency of the aggressive life-sustaining support subset (from 15.3% to 9.8%, *p* = 0.070) and a modest increasing tendency of the withdrawal subgroup (from 2.1% to 4.7%, *p* = 0.127).

**Fig. 4:**
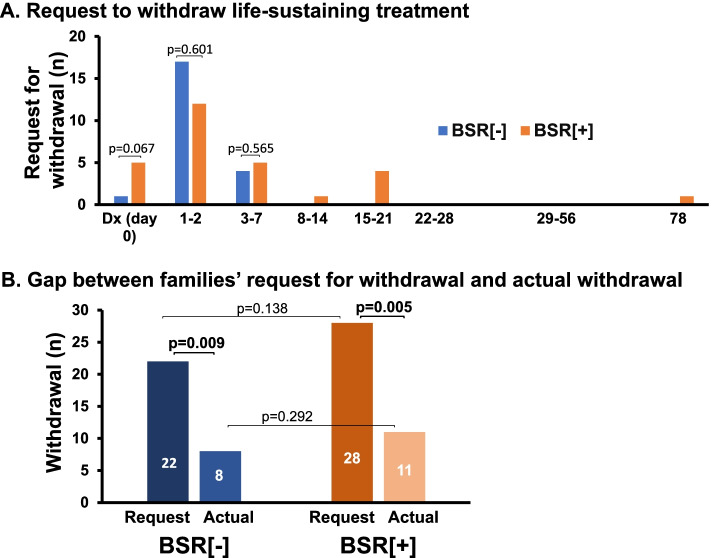
Temporal changes in the number of requests to withdraw life-sustaining treatment and actual withdrawal. The numbers of the requests of patients’ families to withdraw life-sustaining treatment after specific days of diagnosis (Dx) on neurological status were shown (**A**). Total numbers of families’ requests for withdrawal reached 22 cases in brainstem reflex [-] (BSR[-]) and 28 cases in BSR[ +] group (**B**). Physicians, however, actually withdrew the life-sustaining treatment in only 8 and 11 cases in the BSR[-] and BSR[ +] group, respectively

Figure 4A illustrates serial changes in the number of the requests of patients’ families/surrogates to withdraw life-sustaining treatment during hospitalization. Most of the requests were made within 7 days from the initial diagnosis on patients’ neurological status although several families requested withdrawal of treatment after 15 days, particularly those of patients with BSR[ +]. When the cumulative number of the requests was assessed, it reached 22 at day 3–7 in the BSR[-] group. In the BSR[ +] group, it gradually increased from 5 to 22 cases at day 3–7 (*p* < 0.001), and finally to 28 cases at day 78 (*P* < 0.001). Despite considerable numbers of the families’ requests, physicians actually withdrew the life-sustaining treatment in only 8 cases with BSR[-] (*p* = 0.009) and 11 cases with BSR[ +] group (*p* = 0.005, Fig. 4B).

Withdrawal/withholding of life-sustaining treatment included discontinuation of drugs (catecholamines, antibiotics), enteral feeding and hemodialysis (Table 3). Catecholamines were withheld in 69.8% of the patients with BSR[-] (vs. 40.8% in BSR[ +], *p* < 0.001). Tracheostomy was performed in more patients with BSR[ +] than in those with BSR[-] (27.4% vs. 0.8%, *p* < 0.001). For withdrawal of life-sustaining treatment, endotracheal extubation was conducted in 3 patients with BSR[ +], and maintenance hemodialysis was discontinued in 5 cases with BSR[ +] and one with BSR[-].

**Table 3:**
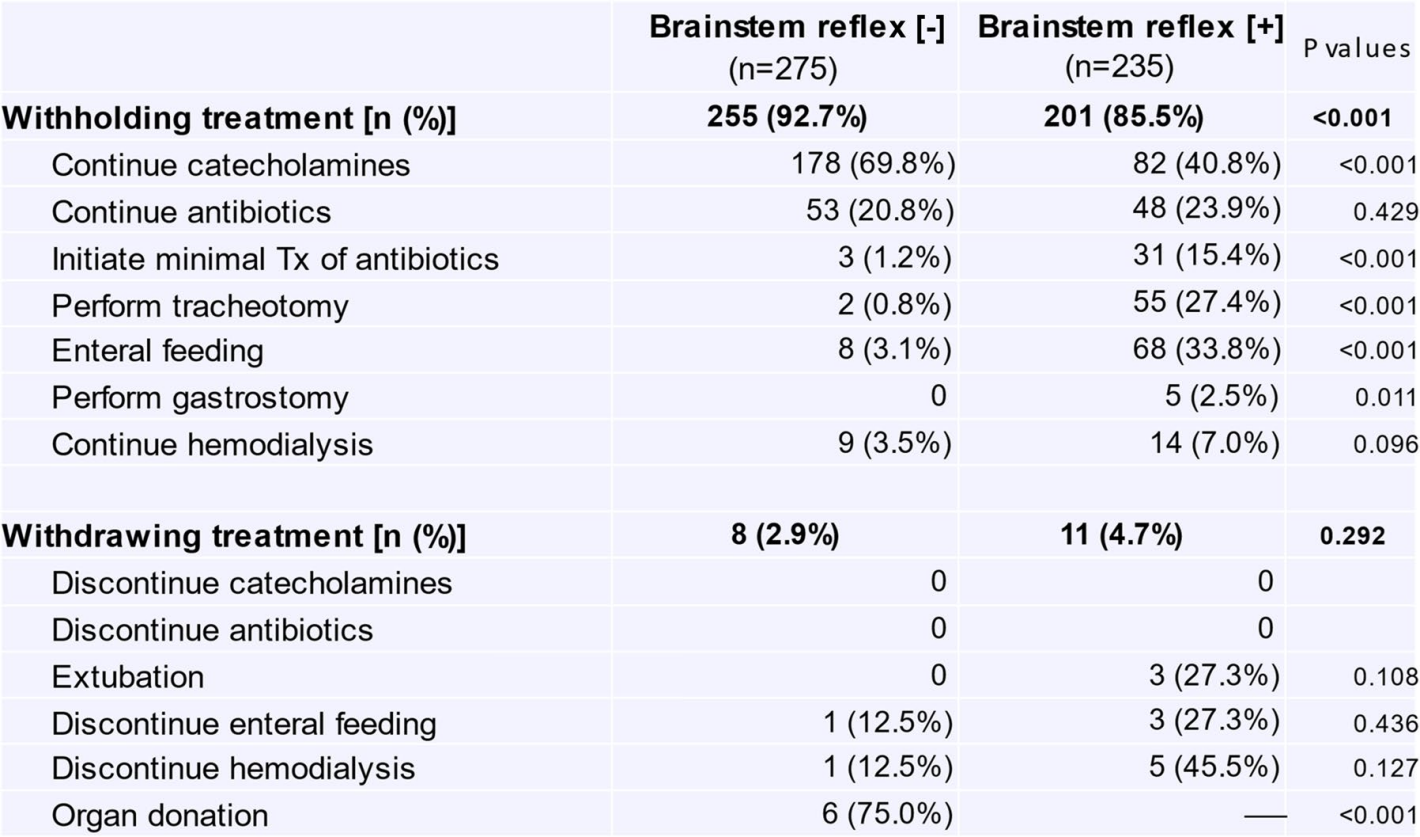
Withdrawal or withholding of life-sustaining treatment

Finally, the role of physicians’ suggestions and requests of patients’ families/surrogates in the withdrawal from life-sustaining treatment was evaluated. There was observed no difference in the ratio of physicians’ suggestions to families’ requests between BSR[-] and BSR[ +] patients (*p* = 0.251, Table 4A). When each medical practice was evaluated, physicians recommended organ transplantation following the withdrawal of life-sustaining treatment, particularly in BSR[-] patients (*p* = 0.022) and preferred the discontinuation of hemodialysis in BSR[ +] patients (*p* = 0.020, Table 4B). In contrast, patients’ families requested the discontinuation of mechanical ventilation in both BSR[-] (*p* = 0.029) and BSR[ +] patients (*p* = 0.022).

**Table 4:**
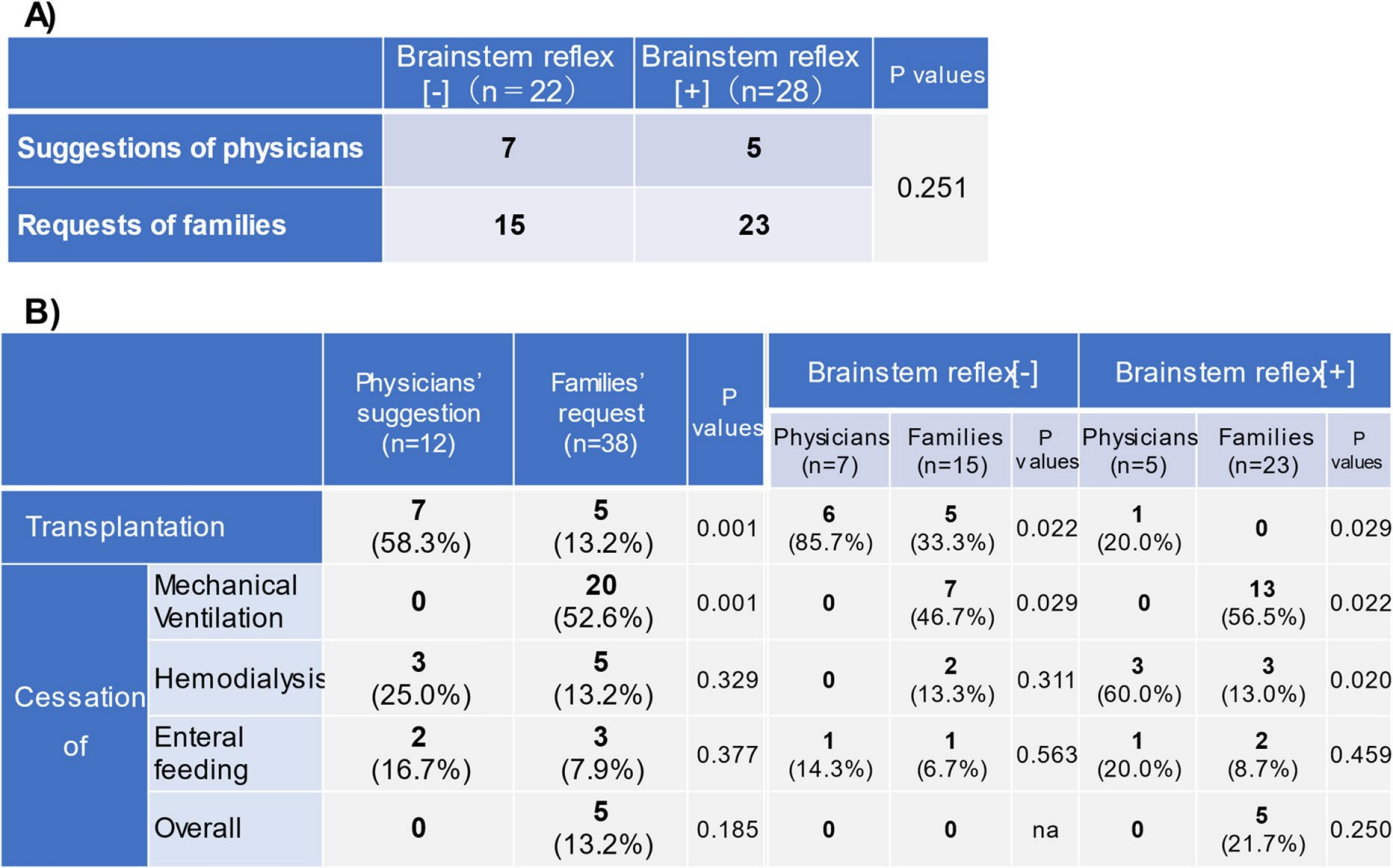
Impacts of physicians’ suggestions and families’ requests on withdrawal policy

## Discussion

Intracranial hemorrhage/cerebral infarction and CPA are serious events that cause ominous sequelae, including severe brain damage with poor neurological prognosis and/or brainstem dysfunction. There have been reported many studies and reviews showing a substantial number of patients who develop severe brain damage following intracranial hemorrhage/infarction or resuscitation from CPA [18, 19]. Additionally, in some resuscitated patients, irreversible hypoxic encephalopathy ensues which requires differential diagnosis for brain death. Such devastating status always worries physicians with their attitudes toward treatment policies because interrupting ongoing therapies might evoke ethicolegal and religious issues, particularly in Japan and other Asian countries [4, 5, 8, 10]. To the extent that withdrawal of treatment and execution of organ donation depend on the brain status of patients, it appears extremely critical to recognize the characteristics and the temporal profiles of patients following brain hemorrhage/infarction or resuscitated from CPA.

A. ***Characterization of patients with BSR[-]/[ +]***
  The present study shows that during the last 11 years, we experienced 510 patients who suffered severe sequelae of brain damage resulting from intracranial hemorrhage/infarction and CPA in our two affiliated hospitals (Fig. 1). These unconscious patients were classified as cases with absent brainstem reflex (i.e., BSR[-]) and those with the preserved reflex (i.e., BSR[ +]). Thus, 53.9% of the patients developed severe brain damage with BSR[-], a status conventionally deemed as a category of ‘brainstem death’ [20–22] and resulted in markedly less survival; the patients of this subgroup had survival rate of 0.7% and LOS of 3 days (Table 2), as compared with the patients with BSR[ +] showing 31.9% of survival rate and 16 days of LOS. These findings are consistent with the premise that brainstem dysfunction reflects more severe brain injury [15] and may further lend support to the presumption that impaired BSR affects not only the physicians’ attitudes toward treatment practices but also the decision-making process of patients’ families; they might request to withdraw life-sustaining treatment and propose organ donation. Indeed, organ transplantation was performed in six cases (Table 3).
  The impact of brainstem function and EEG activity on mortality remains a matter of controversy. The present study showed that among patients with BSR[-], only 29.8% of the patients underwent EEG examination, and most of this subgroup manifested a flat EEG (Fig. 2). Furthermore, because of a paucity of survivors in the BSR[-] group, the survival rate did not depend on whether an EEG was implemented (*p* = 0.088) nor on the types of the EEG wave pattern (*p* = 1.0). In this regard, several statements and reports indicate that EEG is not a requirement for the diagnosis of brain death unless mandated by regional laws [23, 24]. It turns out therefore that implementation of the EEG plays a permissive role in patients with BSR[-].
  In contrast, a substantial number of the patients with BSR[ +] had a chance to undergo EEG examination (i.e., 69.4%, Fig. 2), probably due to a longer period of hospitalization. Among this subgroup, there was no difference in survival rate between patients with flat EEGs (29.2% [= 19/65]) and those with diffuse slow waves (36.0% [= 31/86], *p* = 0.378). Because a number of the patients with BSR[ +] had no chance to undergo EEG examination, it awaits further evaluation whether EEG findings affect survival rate in patients with BSR[ +].
B. ***Treatment policy***
  Since the current study shows an intimate association between brainstem activity and the prognosis of patients with severe brain damage, the distinct neurological properties between BSR[-]- and BSR[ +]-patients may influence the treatment policies and attitudes of physicians. At the time of initial diagnosis on neurological status, most families of the patients with BSR[-] and BSR[ +] had willingness to withhold life-sustaining treatment (Fig. 3). In patients with BSR[-], however, the ratio of the patients receiving aggressive life-sustaining treatment was less than in those with BSR[ +] (6.9% vs. 15.3%, *p* = 0.002). This difference could be ascribed to the impact of impaired brainstem function [15]. When temporal factors (i.e., interval between the diagnosis and actual implementation of treatment policies) were taken into consideration, there existed no serial changes in the proportion of each treatment policy (i.e., aggressive, withhold and withdraw) in patients with BSR[-] (*p* = 0.432). In patients with BSR[ +], by contrast, the changes in the proportion nearly attained significance (*p* = 0.072), probably due to an additive impact of a decrease in cases with aggressive life-sustaining support (from 15.3% to 9.8%, *p* = 0.070) and a modest increasing tendency in the patients who had withdrawal of life-sustaining treatment (from 2.1% to 4.7%, *p* = 0.127). These distinct effects could be associated with longer hospitalization in BSR[ +] cases (i.e., 16 days vs. 3 days, for BSR[ +] and BSR[-], respectively, *p* < 0.001).
  The implications of withdrawal or withholding of treatment in end-of-life care may be affected by physicians’ attitudes toward life-sustaining treatment. Vincent [25] previously showed in a questionnaire survey conducted in 16 European countries that 93% of the respondents withheld treatment from patients with no hope of a meaningful life. A recent questionnaire survey on the treatment attitude of physicians in Asia demonstrated that they often withheld but seldom withdrew life-sustaining treatments in ICU patients [4]. This study included 1465 respondents, among whom 224 physicians were enrolled from Japan. The study showed that 70.2% of respondents withheld and 20.7% withdrew life-sustaining treatment. When restricted to Japanese physicians, approximately 90% respondents withheld but only 10% withdrew life-sustaining treatment. The trends observed in these findings are hence compatible with the results of our current study. Collectively, these findings observed in Asia lend support to the contention that there is an unacceptable social background to interrupting medical practice. Close communication between physicians and families and in-depth discussion would offer more fruitful end-of-life care.
  The present study shows a striking discrepancy in number between the requests from patients’ families to withdraw treatment and the actual withdrawal by the physicians. Thus, the cumulative number of requests to withdraw life-sustaining treatment increased during a couple of week period (BSR[-]; from 1 to 22 cases, BSR[ +]; from 5 to 28 cases, Fig. 4). Nevertheless, actual implementation of the withdrawal was much less in both groups (8 and 11 cases for BSR[-] and BSR[ +], respectively). The discord between these results may be accounted for not only by personal attitudes of physicians toward treatment policies but also by religious belief of them. Indeed, Asian physicians, including Japanese, tend to desist from discussing with families the withdrawal of ongoing life-sustaining therapy [4, 5], which might discourage the families from requesting withdrawal of treatment. Furthermore, physicians’ decision may be affected by the prognostic uncertainty of patients. The BSR[ +] patients had longer length of hospital stays than those with BSR[-], but nearly the same withdrawal ratio (Figs. 3, 4), which could reflect the suspended decision-making on withdrawal of life-sustaining treatment. Finally, ethicolegal circumstance in Japan may deter physicians from interrupting life-supporting treatment. Although the guidelines by the Government and several academic societies qualified the withdrawal/withholding of treatment as end-of-life care practices [11–14], our physicians might hesitate to implement justifiable withdrawal to avoid lawsuits. This important issue should be more thoroughly recognized, and successful end-of-life care needs to be established with nationwide consensus endorsed by ethicolegal frameworks.
  Of note, the present study shows that there are some differences in the suggestions and requests for withdrawal between physicians and patients’ families. As illustrated in Table 4, physicians suggested withdrawal of life-sustaining treatment, resulting in implementation of organ transplantation, particularly in patients with BSR[-]. In striking contrast, 20 families/surrogates of the patients requested the discontinuation of mechanical ventilation whereas none of the physicians had suggestion for this withdrawal. Unlike European physicians, Japanese doctors are less likely to withdraw life-sustaining treatment [4, 5], which attitude may be reflected by no suggestion regarding the cessation of mechanical ventilation. Alternatively, the physicians essentially recognize that the recommendation of organ transplantation is rendered contributory to transplant therapy and may offer an opportunity for functional restoration to many potential recipients with end-stage organs. The attitudes of Japanese physicians may thus constitute a determinant of end-of-life care policies that cannot be modified so easily probably because of our religious belief or societal culture.
C. ***Limitations***
  The present study has been conducted in two affiliated institutions. Thus, the critical care management and ethical responses to CPA in these two institutions may not be fully identical. Nevertheless, communication between the staff of these two facilities is well maintained through regular assembly and medical and other technical information is shared, which would minimize the gap between two hospitals. Caveat is in order, however, since the attitudes of our medical staff toward treatment policies might be deviated from a national consensus on end-of-life care. Finally, life-sustaining treatment policy for patients with severe brain damage may be affected by personal attitudes of physicians toward treatment of which physicians’ religious belief could be a determinant. Reinforced decision-making frameworks involving not only physicians but also co-medical staff and lawyers would hence unravel this problem.

## Conclusions

The present study demonstrated the characteristics of the patients who suffered severe brain hemorrhage/infarction or were resuscitated from CPA but with poor neurological outcomes. Preserved BSR constitutes a determinant of survival and length of hospitalization. More importantly, although requests of patients’ families to withdraw life-sustaining treatment increased over time, actual interruption does not correspond to the request due to multifaceted confounding factors, including medicolegal frameworks and physicians’ attitudes to the treatment. This discrepancy appears to be more conspicuous in Japan, and sociolegal, ethical and religious issues surrounding our medical circumstance should be taken into consideration when discussing end-of-life care for patients with severe brain damage with poor neurological prognosis.

## Data Availability

The datasets used and/or analyzed during the current study are available from the corresponding author (Haruaki Wakatake: haru_waka_2110@marianna-u.ac.jp) on reasonable request.

## Abbreviations

CPA: Cardiopulmonary arrest
OHCA: Out-of-hospital cardiac arrest
BSR: Brainstem reflex
ICU: Intensive care unit
HCU: High care unit
NCSE: Non-convulsive status epileptics
LOS: Length of stay
